# A comprehensive review on ecology, life cycle and use of *Tecoma stans* (bignoneaceae)

**DOI:** 10.1186/s40529-024-00412-4

**Published:** 2024-02-13

**Authors:** Simrat Singh, Chad Thomas Miller, Parminder Singh, Rishu Sharma, Nepu Rana, Ashok Kumar Dhakad, Rajesh Kumar Dubey

**Affiliations:** 1 Department of Floriculture and Landscaping, Punjab Agricultural University, Ludhiana, India; 2 Department of Horticulture and Landscape Architecture, Colorado State University, Fort Collins, CO USA; 3 Department of Forestry and Natural Resources, Punjab Agricultural University, Ludhiana, India

**Keywords:** *Tecoma stans*, Distribution, Ecology, Pollination, Reproductive biology, Invasiveness

## Abstract

*Tecoma stans* is a widely distributed tall ornamental shrub in the plains of Indian subcontinent and is considered an invasive species across Argentina, Australia, South Africa, Pacific Islands and tropical regions of Asia. Besides having an ornamental significance, *T. stans* has been extensively investigated for its pharmaceutical applications as a source of bioactive compounds. In addition, the shrub is cultivated commercially as a potted flowering plant. We believe that *T. stans*, being a hardy, invasive and aggressively growing species, holds a considerable potential and a promising solution for re-greening waste and degraded lands outside its invasive range, due to its wider adaptability and drought tolerant characteristics. The shrub is an excellent source of pollen and nectar, that attracts diverse insect-pollinators and several species of birds. The prudent plantation of this shrub has the potential to restore the ecology of barren landscapes, that can change its perspective of ‘being invasive’ to ‘being ecologically healthy’ across the tropical, semi-arid and subtropical regions worldwide. This paper reviews the current updates on ecology, life cycle including morphology, plant growth characteristics, flowering phenology, reproductive biology, breeding system and fruiting of *T. stans*. In addition, details on insect-pollinator diversity and natural regeneration potential have also been discussed, besides highlighting its therapeutic and landscape use.

**Supplementary Information:**

The online version contains supplementary material available at 10.1186/s40529-024-00412-4.

## Introduction

*Tecoma stans* (L.) Juss. ex Kunth is perennial shrub belonging to the Bignoneaceae or trumpet vine family, which is comprised of more than 100 genera and 600 species (Bor and Raizada 1990). The species, over time has had several nomenclature authorities, however the current recognized nomenclature is *Tecoma stans* (L.) Juss. ex Kunth (IPNI 2023). It is a widely distributed non-native shrub in the Indian subcontinent plains, inhabiting extensively in the semi-arid, tropical to subtropical regions. Due to its excellent adaptation, it has also naturalized in lower and mid Himalayan ranges. *Tecoma* is derived from Mexican term ‘*Tecomaxochiti’*, meaning ‘vessel flower’ as observed by the cup or trumpet-shaped blooms and ‘*stans*’ symbolizes ‘standing’ or ‘erect’ (Bhat 2019), as evident from the erect growing shoots emerging from arching branches. The South American native is commonly known as ‘yellow trumpet bush’, ‘yellow bells’, ‘yellow elder’ ‘Ramat Emas’, and ‘Ginger-Thomas’ (CABI 2023). The *Tecoma* flower is considered as an official emblem of the United States Virgin Islands and Bahamas. Due to its recurring bright yellow trumpet shaped flowers and persistent and dark green foliage round year, it is extensively used for landscape beautification. In addition, *Tecoma stans* is a source of bioactive compounds that are extracted from fruits and flowers. Several studies (Sbihi et al. 2015; Taher et al. 2016; Bakr et al. 2019; Khatak et al. 2019) have assessed potential pharmacological uses of *T. stans* with therapeutic significance. The genus *Tecoma* is believed to be a taxonomically diverse group, yet revealing similarity in several of the polymorphic characters (leaves, branching pattern and inflorescence form) believed to be influenced by the environment. To date, a comprehensive assessment of ecological distribution and diversity of *T. stans* is deficient in the literature. Moreover, the morphology, reproductive biology, growth pattern and regeneration potential, have not been reviewed comprehensively. In this review, the description of 16 *Tecoma* species has been presented in tabular format (supplementary table S1), to acknowledge the readers for appropriate distinction between these species based on vegetative and floral traits. The present review highlights these aspects citing classical and recent references, dating between 1864 to 2023. Further, the review presents a discussion on functional importance of *T. stans* in the landscape and its daptability and response to climate change impacts. Seasonal patterns of flowering and fruiting have also been highlighted, along with a detailed account of insect-pollinators documentation.

## Ecological distribution and diversity

**Fig. 1:**
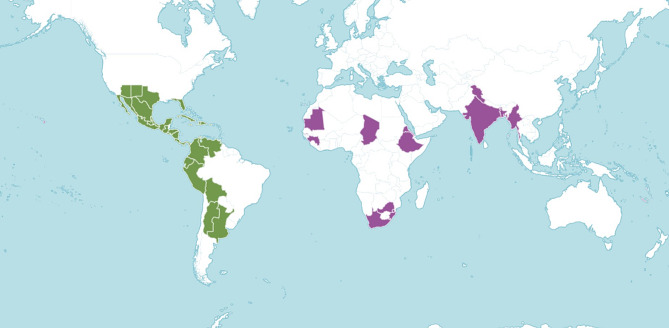
Distribution of *Tecoma stans* in its native (green) and introduced (purple) range (KBD 2023)

The genus *Tecoma* inhabits drier regions at an altitude of 0-2000 m above sea level in areas receiving mean annual rainfall of 600 to 1100 mm and temperature in range of 20 to 32^o^C (Orwa et al. 2009). *Tecoma* has a wide natural distribution in neotropical geographical regions comprising Central and South America, including the tropical southern part of Mexico and the Caribbean (Fig. 1). The species is reported to be introduced in 86 countries and islands (GBIF 2023). The genus is stated to be of least concern (IUCN 2019) and not threatened (BGCI 2019) comprising 16 species, out of which 14 species have been found native to Neotropics and two in Africa (Gentry 1992; POWO 2023). The shrub inhabits areas with abundant sunshine, on a well-drained low to medium fertility soils. Predominately, the shrub is found in coastal and inter-Andean regions’ (Macbride 1961), however, isolated populations of *Tecoma* have also been documented by several explorers [(Bridgewater et al. 2003); (Pennington et al. 2000, 2004a; Prado and Gibbs 1993; Wood 2006), in the regions adjoining seasonally dry forests of South America. However, the shrub is considered as an exotic species in neo-tropical regions comprising Central America, Caribbean and South America. *Tecoma* species commonly found in northern parts of India include *T. stans*, *T. capensis, T. gaudichaudi* and *T. smithii*.

The genus *Tecoma* comprises two basic species, *Tecoma fulva* ssp. Garrocha (Hieron.) J.R.I. Wood and *Tecoma stans (L.) Juss. ex Kunth.* exhibiting distinct floral phenotypes with different species of floral visitors and pollinators. *Tecoma stans* flowers are chiefly characterized with yellow flowers and are specifically pollinated by hummingbirds. The flowers of *Tecoma fulva* ssp. Garrocha has tubular red-orange flowers that are bee pollinated (Curti and Ortega-Baes 2011). The hummingbird-pollinated group is limited to the regions adjoining central Andes, from Peru to northern Chile and Argentina, and appears to be undergoing an active evolutionary divergence with appearance of similar morpho-types in inter-Andean valleys. The later yellow-flowered group consists of several polymorphic species varying in their leaf architecture and form. The one with simple leaves inhabit in western Ecuador, second with tri-foliate leaves is common in northwestern Peru and southwestern Ecuador, and third morpho-type with slightly more obtuse leaflets are found at higher altitudes in the Andes of Peru and Bolivia.

## Invasiveness

The potential encroachment of *T. stans* inhabiting waste and degraded lands has been documented by several researchers (Gilman and Watson 1993; Klein 2002) and important biological databases (USDA ARS 2013; GISD 2023; PIER 2023) worldwide (e.g., Atlantic Islands, Asia, Argentina, Australia, South Africa, and Pacific Islands). In Australia, *T. stans* appear as dense shrubs that compete with native species growth and regeneration. The shrub was brought to Brazil as an ornamental plant, but spread quickly throughout pasture-lands and non-crop areas. *Tecoma* can be seen invading roadsides and near watercourses in subtropical and tropical savannah Brazil, where it is considered an invasive species (Cunningham Peter 2008). *Tecoma* plants are regarded as unwelcoming in the Brazilian state of Parana and since 1995 the species has been illegal to cultivate and sell in the nursery. In Namibia, *T. stans* has been found invasive in Outjo region (Macdonald and Nott 1987). This shrub has been categorized as class one invasive alien species in South Africa, to the extent that its vegetation is no longer tolerated in countryside and in urban areas. The trading on its propagules (seeds, unrooted cuttings, suckers, rooted plants) is prohibited and its transport or dispersal to other regions of the country is considered illegal (Henderson 2001). Further, it is believed that foraging of leaves could present a substantial health danger to cattle. Thus, it can be concluded that wide-spread invasion of *Tecoma* could be detrimental for the existence of native species, that eventually may weaken their growth and deprive them with availability of scare natural resources (Klein 2002).

*Tecoma* is an anemochory species, which, disperses its seeds far and wide, lending to a major factor in its invasive tendencies. The ability of *Tecoma* to bear flowers and fruits round the year in diverse agro-climate enables it to produces copious amounts of wind-borne seeds (Gilman and Watson 1993; Giddy 2004). The shrub has the potential to naturally perpetuate vegetatively through root suckers, often seen arising in clusters adjoining the crown of the shrub. Other factors favouring its invasive potential include rapid growth, bearing flowers during second year of its establishment, potential for attracting diverse insect-pollinators, drought tolerance, adaptability to wide range of soils, and its appreciable resistance to pests and diseases. Further, the orthodox seeds, can be stored for long periods without any considerable loss in germination under ideal conditions (Orwa et al. 2009).

## Morphology

*Tecoma stans* can reach up to a height of 8.0 m, but rarely exceed 10 m with stem diameter of up to 25 cm in its natural habitat. The leaf arrangement is opposite, and leaflet margins are serrated. The mature leaves are imparipinnate with usually simple leaf pair at the first node followed by emergence of tri-foliate leaflets arranged in opposite pair. The nodes at the terminus have compound leaves comprised of 5 to 13 leaflet pairs and one terminal leaflet. The leaflets are lanceolate to elliptic, ranging 3.8 to 12.7 cm in length, 2.5 to 5.08 cm wide, and olive green. The leaflets have dentate to serrate margins, longitudinally extended with a broad base and a pointed tip. The thin leaflets of *Tecoma* are considered as a heliophyte and apparently mesomorphic. The leaves are glabrous, appear light green above and paler under the surface. The leaf morphology and the leaflet number vary greatly in different species and is believed to be much affected by the age of plant and environmental factors (Rana et al. 2023). The trumpet shaped bright yellow flowers in *Tecoma* appear in raceme form of inflorescence containing single ovary and many ovules. Its flowers are borne in terminal panicles, with a yellow corolla of 4 to 4.5 cm long and the nectaries are at the base of the ovary. Current season’s growth bear bright yellow funnel shaped flowers in clusters within two years. The yellow trumpet shaped flowers are borne in loose terminal racemes or panicles that are only slightly fragrant (Neal 1948), or have also been reported to be “very fragrant” in Mexico (Vasey and Rose 1890). The variety *velutina*, in contrast, is reported to lack odor (Bailey 1941). The flowers of *T. stans* contain certain flower pigments. Scogin (1980) isolated several floral pigments that include glycosides of cyanidin, delphinidin and pelargonidin.

The stems are glabrous, 4-angled with varying hues of green during young stage turning to pale or reddish brown in previous season’s growth. The young immature stems can be seen with conspicuous whitish elongated lenticels with a grayish corky bark. These lenticels appear oval to round in form with longitudinal fissures, more prominent over older stems that sloughs off readily. As the shrub ages, the bark becomes rough textures and appear fissured. The fruit pods are elongated (11 to 20 cm) and appear compressed. The pods contain about 10 to 20 seeds in each locule and are non-endospermic with papery appearance of seed coat. Axillary buds in leaf axils of shoots are often found superposed in pairs. Fleshy pseudo stipular pair of bud scales, turn brown and scarious before bark matures over the branch. The bark averaged 2 to 3 mm thick develops over mature stems reaching 10 cm in diameter. The wood is moderately dense and heavy with presence of prominent growth rings (Record and Hess 1940). However, it is not certain whether these rings are formed as a result of seasonal or alternating cycles of vegetative and reproductive growth.

### Flowering phenology

The plant is seen flowering and fruiting throughout the year, but with a high proportion flowering during open sunshine with cold to mild temperatures (11.5 to 38.0 °C). Flowering in *T. stans* varies from recurrent flowering to seasonal. In Mexico, *T. stans* flowers profusely between August and November, typically exhibiting two flushes annually (Pesman 1962). In the rugged rainforests of Costa Rica, this species flowers from November to April (Allen 1956), and during September to January in the Republic of Colombia. In Florida, *T. stans* blooms during spring, extending its flowering till September (Apgar 1910). In Puerto Rico, *T. stans* can be seen flowering profusely during winters, although reveal flowers ceaselessly throughout the year (Hume 1951).

On the Indian sub-continent, the species flowers throughout the year, profusely during August through December (Bor and Raizada 1954). Corner (1940), observed that *T*. *stans* flowers several times a year, particularly during rains following a period of dry spells. It was therefore concluded that *Tecoma* responds to sudden drops in air temperature as evident from profuse flush of flowers emerging after rains or pre-monsoon showers. Torres and Lopez (2011a) characterized *Tecoma* as a long day (LD) plant and revealed a direct relation between photoperiod and flowering on longer exposure to far-red light under LDs. In the warmer and tropical regions, the species often tends to bloom profusely during mild winters. The dry season accompanying morning temperature range of 22–25 ^o^C (71.6–77.0 ^o^F) have been found more favorable for availability of abundant pollinators around *Tecoma* shrub, thereby enhancing the probability of pollination and fruit set (Kumar and Singh 1988).

### Palynology and pollination

The basic chromosome number of *T. stans* is *n* = 7, with polyploidy and aneuploid chromosome addition or loss accounting for the current variability (Goldblatt and Gentry 1979). *Tecoma* species flowers cannot set fruits and reproduce with their own pollen due to the presence of dichogamy (Muller 1868). The pollen grains exhibit a distinct tricolfate form with three germinal furrows with symmetrical and globose structure (Gentry 1979). In the tribe Tecomeae, two more pollen types have been identified – one is monocolpate, with a single germinal furrow; and stephanocolpate, with arrangement of germinal furrows around the equator of the grain (Gentry 1979). The pollen grains appear whitish and spherical, with smooth but sticky surface. Only 12 ± 3.0% of the pollen examined were found shriveled while ascertaining their viability through acetocarmine test (author’s personal observation). Suryakanta (1973) commented that the pollen grains of all lianas (climbers with or without tendrils) exhibit a lot of variation regarding both apertures and exine patterns. Climbers from the Tecomeae tribe, like *Pandorea*, did not exhibit such variation in pollen morphology.

The two lobes of stigma separate on being receptive, with the lower lobe curling towards the ventral side of the corolla tube and the upper lobe resting against the dorsal side of the tube. It stays that way as the bloom ages, with the lowest lobe curling unless it is touched. The majority of the time, pollinator activity in the corolla tube would act as a stimulus for the closure. Studies reveal that the stigma will regain its position if the closure was caused by contact (Newcombe 1922, 1924; Petersen et al. 1982). When foraging on *Campsis radicans*, ruby-throated honey eaters have been found to touch the anthers or stigma by probing inside deep in the corolla tube, but when they probed between the calyx and corolla, they had no influence on pollination (Bertin 1982). Harborne and Smith (1978) concluded that bee-pollinated flowers usually have a delphinidin-dominated floral pigments, while those pollinated by lepidopteran insects predominately have cyanidin-based pigments.

### Pollinator diversity

**Fig. 2:**
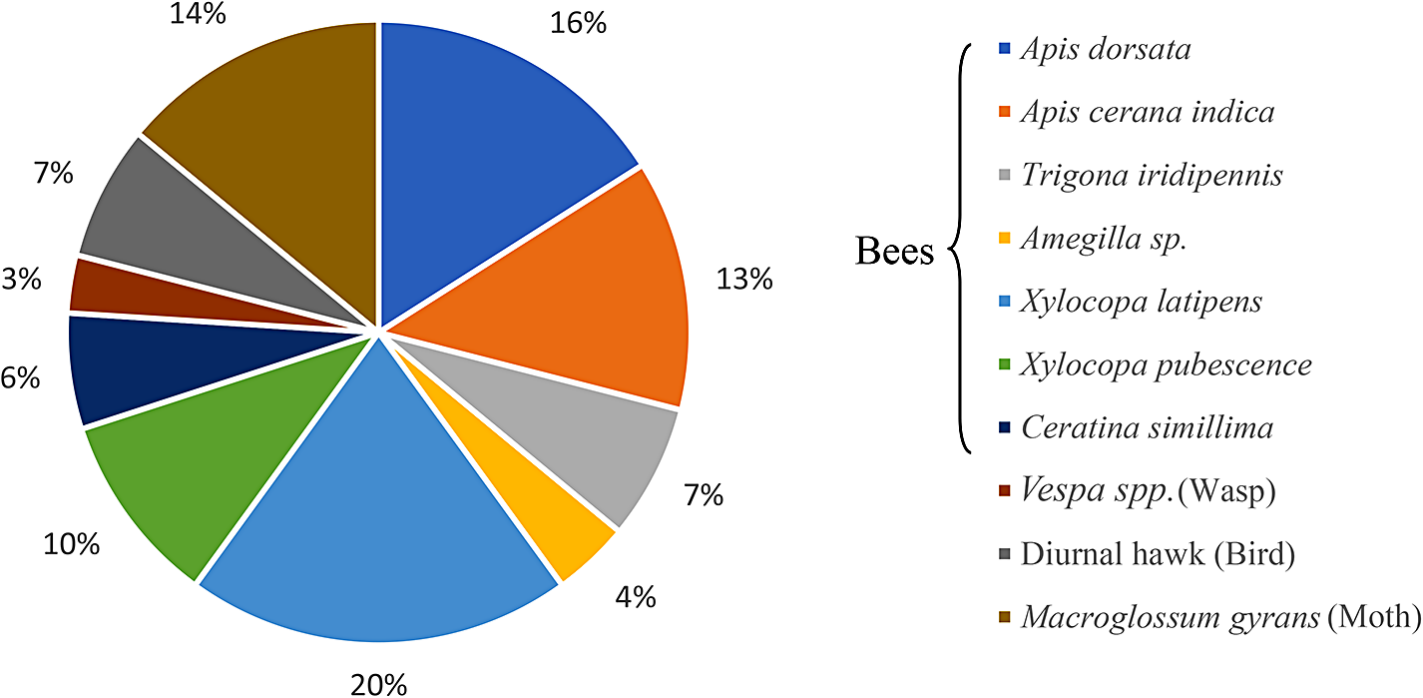
Percent proportion of foraging species on *Tecoma stans* Reconstructed from tabular data, (Adapted from Menninger 1962)

The flowers of *T. stans* are reported to be visited by several insect species (Fig. 2) such as bees, butterflies, and moths and by hummingbirds (Menninger 1962). The flowers are an important forage resource for the Apoidea family to around 48 species of bees (Silva et al. 2007). Around ten insect taxonomic groups have been reported in north western Costa Rica with a specific foraging period (Table 1, Wojcik 2011). Amongst several pollinators, Jamaican Mango hummingbirds were observed to visit flowers often, compared to the visitation by Honey bees (*Apis mellifera* L., family: Apidae; Order: Hymenoptera) and other insects. The nectar of *Tecoma* flower has been reported ‘nutritious food’ for the foraging bees (Standley 1926). The size of bee species *A. mellifera* was found too small to come in contact with the anthers or style effectively for ensuring successful pollination. *Tecoma stans* is considered primarily a hummingbird-pol1inated flowers and it seem structurally well-suited for conducting effective transfer of pollen ensuring pollination success. Bee species such as *Centris tarsata* Smith and *Exomalopsis fulvofasciata* Smith (order-Hymenoptera; family-Apidae) are believed to be the effective pollinators found abundantly during the flowering, while *Scaptotrigona depilis* Moure of same order and family was seen as a frequent visitor for robbing nectar and pollen. Nearly 87.5% of the insect species visited *Tecoma* flowers exclusively for foraging nectar, varying in sugar concentration, higher during the day time relative to noon when the nectar gets evaporated. A nectar concentration ranging 26.4–32.7% was quantified at different stage of flowering during 10 am to 2 pm. In addition, the specialized structure called ‘osmophore’ was also detected in the petals (Pelton 1964) that is believed to emit a mild characteristic odor from petals of *Tecoma* during early morning hours.

Wasps such as *Xylocopa latipens, X. pubescence, Vespa sp*. and *Macroglossum gyrans* forage on nectar; all other bee species forage on both pollen and nectar (Bor and Raizada 1990).

**Table 1:** Dominant foraging period of 10 taxonomic insect groups visiting resources of *Tecoma stans. * (adapted from Wojcik 2011)

**Dominant foraging period**		
Early morning	Full day	Sporadic
**Taxonomic groups**		
*Apis*	Centris*	*Epicharis*
*Euglossa*	*Halictus*	*Eulema*
*Mesoplia*	*Trigona*	*Melitoma*
*Xylocopa*		

* *Centris eurypatana* Snelling was the dominant species collected across all three landscapes, constituting 46.8% of all bees collected

### Floral larceny

*Tecoma* flowers yield a sweet nectar in minute quantities in the shallow cupuliform hypogenous disk found at the flower base subtending the ovary. The wide-mouth cone-shaped corolla, gradually narrows down at the base and provides a safe passage for a variety of insects and bird pollinators to reach the nectar. Floral larceny may affect the reproductive success, however, the consequences of foraging of nectar by bees and wasps are sometimes neutral, or may likely reward the flower with successful pollination of the stigma. A Bombax bee (*Genus species*) was found to be a regular visitor at the base of the corolla slits (Bertin 1982). Ants and hummingbirds regularly puncture the corolla base in search of sweet nectar, however, the reward for nectar is reciprocated by repelling the herbivores that usually get annoyed by the presence of these ants (author’s personal observation). Other frequent nectar robbers of *Tecoma* flowers include bees, hawk moth and wasps. *Tecoma stans* often has low natural fruit set, which necessitates the intervention of pollinators to increase the chances of pollination and nectar robbers are believed assist in increased fruit set. However, the studies conclude that *T. stans* planted as an ornamental plant in urban areas had higher probability of resource theft (nectar and pollen) and lower fruit set (Curti and Ortega-Baes 2011). Besides, the structural modifications in floral organs may also discourage frequent visits by small insects for foraging nectar. The inner surface of corolla lobes is slightly sticky, and the base of the filaments are found glandular and pubescent. The calyx also possesses extra-floral nectaries, which are depressed multicellular with saucer-shaped glands (Seibert 1948; Govindu 1950). These glands exude liquid droplets that attracts ants and honeybees. The extra-floral nectaries probably evolved as an adapting strategy that prevent browsing by herbivores. It was observed that this localized pattern is irrespective of nectar production by *Tecoma* flowers in urban and countryside areas, since the secretion pattern was found similar in both the habitats (Arceo-Gomez et al. 2011).

### Reproductive biology

**Fig. 3:**
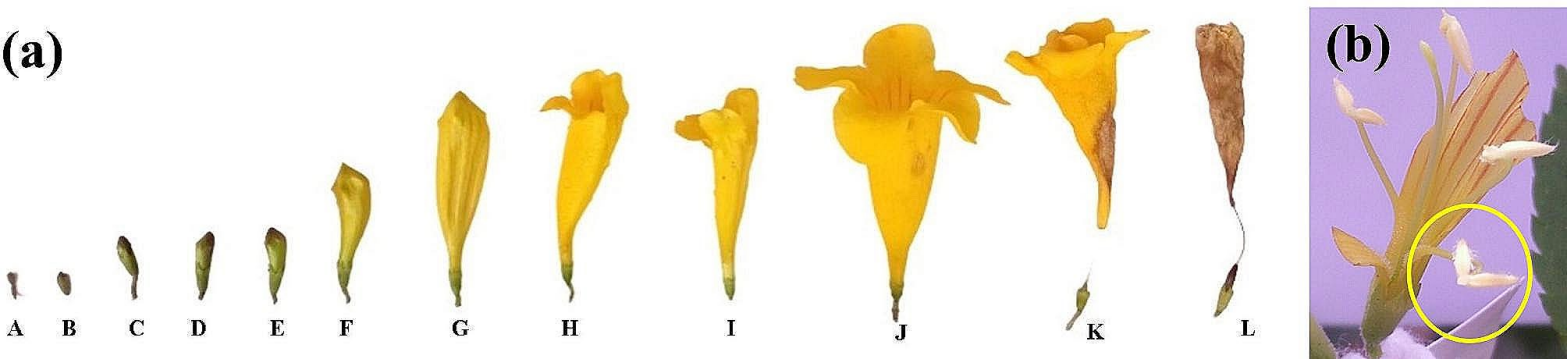
**(a)** Developmental stages in flower of *T. stans. ***(A)** Bud primordia **(B)** Tight bud enclosed within calyx. **(C-E)** Buds at color show stage **(F)** Buds longitudinally and laterally revealing varying hues of yellow. **(G)** Balloon stage **(H-I)** Petal lobes began to unfurl. **(J)** Anthesis **(K-L)** Petals shrivel post pollination and fertilization success. **Scale bars:** A-F: 2 mm; G-I: 40 mm; J-L: 60 mm. **(b)** Two anther lobes begin diverging apart (encircled) during anther dehiscence. (Rana et al. 2023;Photograph cited with permission from Elsevier, license number 5,602,561,230,437)

Understanding the reproductive biology of flowers is crucial to estimate reproductive success through a flower’s breeding behavior and pollinator-flower interaction, which can be variable over changing seasons (Singh 2023). Sexual reproduction is a natural phenomenon to create variation in the progeny and ensures survival of species in adverse climate (Moza and Bhatnagar 2007). The reproductive biology of *T. stans* has been nicely presented (Pelton 1964) and has been recently revised extensively (Rana et al. 2023), incorporating peculiar inflorescence characteristics, various stages of flowers and pod development. Various growth stages were designated (Fig. 3, a) from floral bud emergence till flowering (A to D) and thereafter from fruit set until pod maturity (E to H). The floral buds of *Tecoma* took approximately four days to pass from stage A (bud appearance) through stage C (balloon stage). Anthesis occurred two more days after stage C to the fully open flowers (stage D). The colour variation in corolla was also observed with varying hues of green to yellow with progression of stages A to D. The corolla colour revealed blend of yellow with green during transition between stages B and C.

Fully open flowers revealed four functional stamens that lie bunched in two pairs against the upper lip of the corolla, with one pair slightly visible above the other. The anthers appear villous with tiny hair like projections that facilitates pollen adherence upon dehiscence, thereafter aiding in pollen adhering to pollinator legs and appendages (Curti and Ortega-Baes 2011). During bud anthesis, the two lobes of each anther begin diverging, until the divergence approaches 180 degrees (Fig. 3, b). The pollen sacs of one another align in a straight line orienting parallel and adjacent to the style. The upper pair of anthers dehisce by a longitudinal slit, coinciding with the time of opening of the corolla. Presence of peculiar but a functionless staminode can also be seen that aid in attracting pollinators in guiding their way inside the throat of flower besides playing a structural role in preventing self-pollination (Walker-Larsen and Harder 2000).

The style and stigma protrude towards the upper lip of the corolla. The stigma consists of two large flat lobes with very delicate inner surfaces, which close together at a slight mechanical disturbance or a gentle touch. The lobes appear greenish and are minutely hairy from inside and have smooth outer surface. These sensitive lobes close within 10–30 s after a slight mechanical stimulus and regain their position after 5–10 min. This thigmotropic behavior of stigma ensures cross-pollination by receiving the pollen from pollinators entering the corolla, but prevent self-pollination by closing before the pollen of same flower adhering to the body of pollinators can dust over the receptive stigmatic surface (Pelton 1964).

### Breeding system

**Fig. 4:**
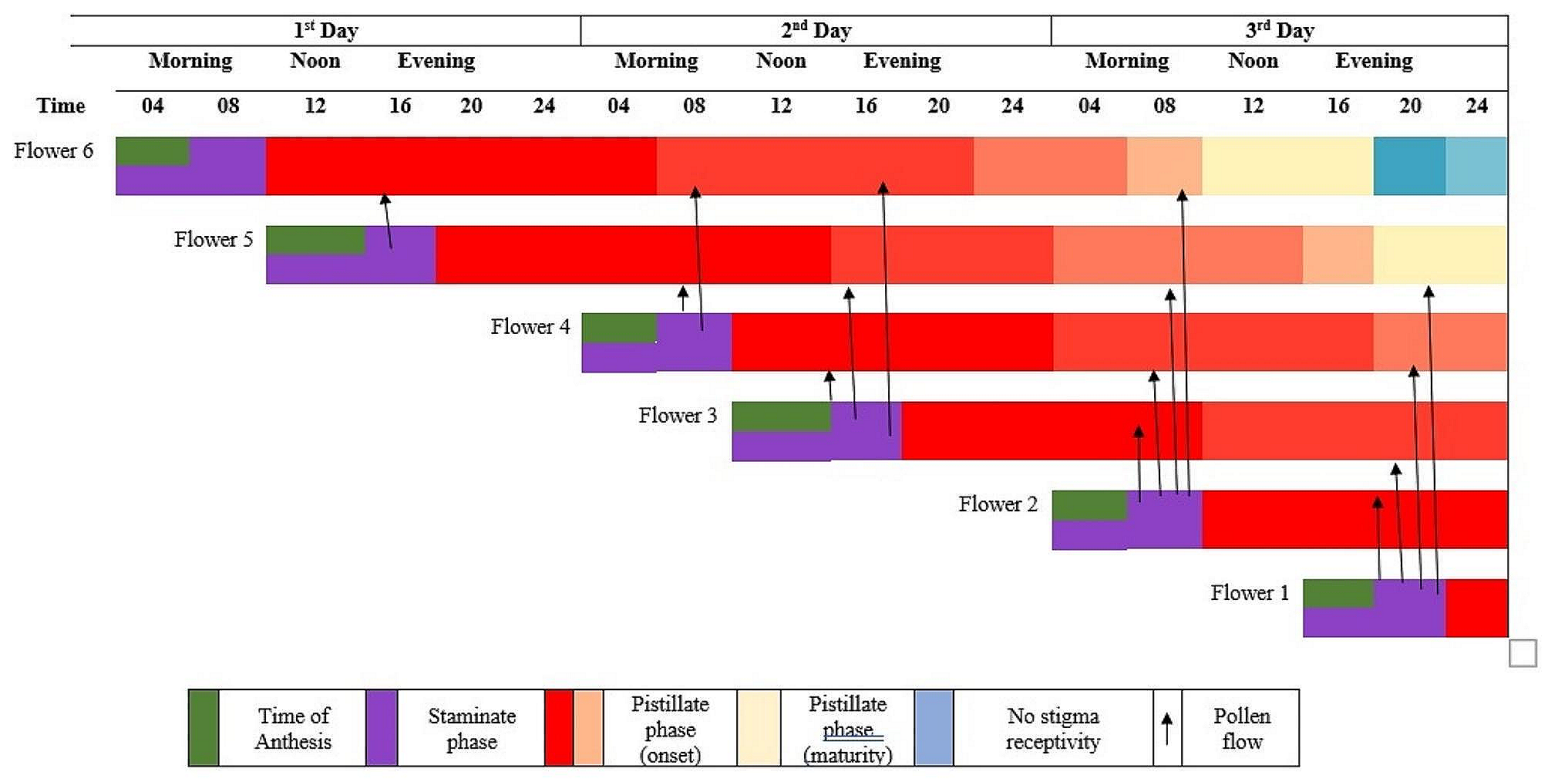
Temporal variation in flow of pollen and its dispersal over other flowers in *Tecoma stans*. Adapted and reconstructed from Rao et al. 2005

*T. stans* exhibits dichogamy however, investigations undertaken (East 1940) revealed the occurrence of protandry in several species from Bignoniaceae. It has been found that self-incompatibility occurs in several unnamed *Tecoma* species (Pelton 1964). The receptivity of only the upper stigmatic surfaces restricts autogamy of open flowers. The chances of self-pollination are also rare, since usually the lobes of stigma are closed in an opened buds and the anthers can only dehisce until the complete flower opening till the third day of anthesis. After a gap of 8 h, the lamellate stigma lobes unfold indicating the commencement of receptivity to the conspecific pollen. Stigma receptivity remains until the early afternoon of the 3rd day in the flowers that open in the morning period and until late night of the 3rd day in the flowers that open in the afternoon period. The duration of stigma receptivity was confirmed by hydrogen peroxide (H_2_O_2_) test (Dafni 1992). The stigma lobes that fail to receive pollen after the receptive period, fall off on the 4th day of anthesis. This indicates that the staminate and pistillate receptivity functions are temporally off-set to exclude self-pollination. Stigmas of morning flowers have the possibility of receiving pollen from the flowers open in the afternoon of the same day, the next day and on the morning of the consecutive day while those of afternoon flowers receive pollen from the flowers that open on the next two consecutive days (Fig. 4). Therefore, it can be concluded that the floral sexual system evidenced in *T. stans* represents a temporal dioecism. This sexual system impedes autogamy, minimizes geitonogamy and enhance chances of xenogamy (Cruden 1977). A hybridization attempt was conducted between several interspecific crosses between *T. stans* x *T. garrocha* with an objective to develop hybrids with potential ornamental value. The selected progeny was found to have an orange corolla with an intermediate flower size with horizontal and vertical diameter of petal limb measured 35.3 mm and 31.4 mm respectively. The length and maximum diameter of petal tube was recorded 43.9 mm and 11.5 mm respectively. The new hybrid had the potential to compete with the Japanese flower markets (Kobayashi et al. 2004).

### Fruiting and seed set

**Fig. 5:**
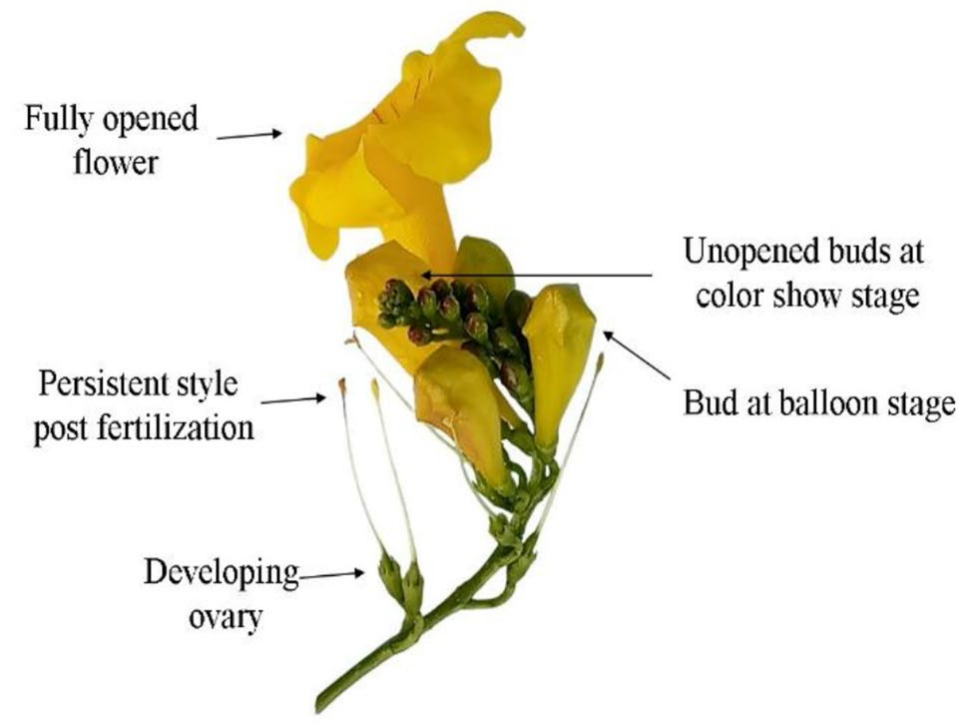
Persistent style with shriveled stigma post fertilization of *Tecoma stans*. Rana et al. 2023; Photograph cited with permission from Elsevier, license number 5,602,561,230,437

**Fig. 6:**
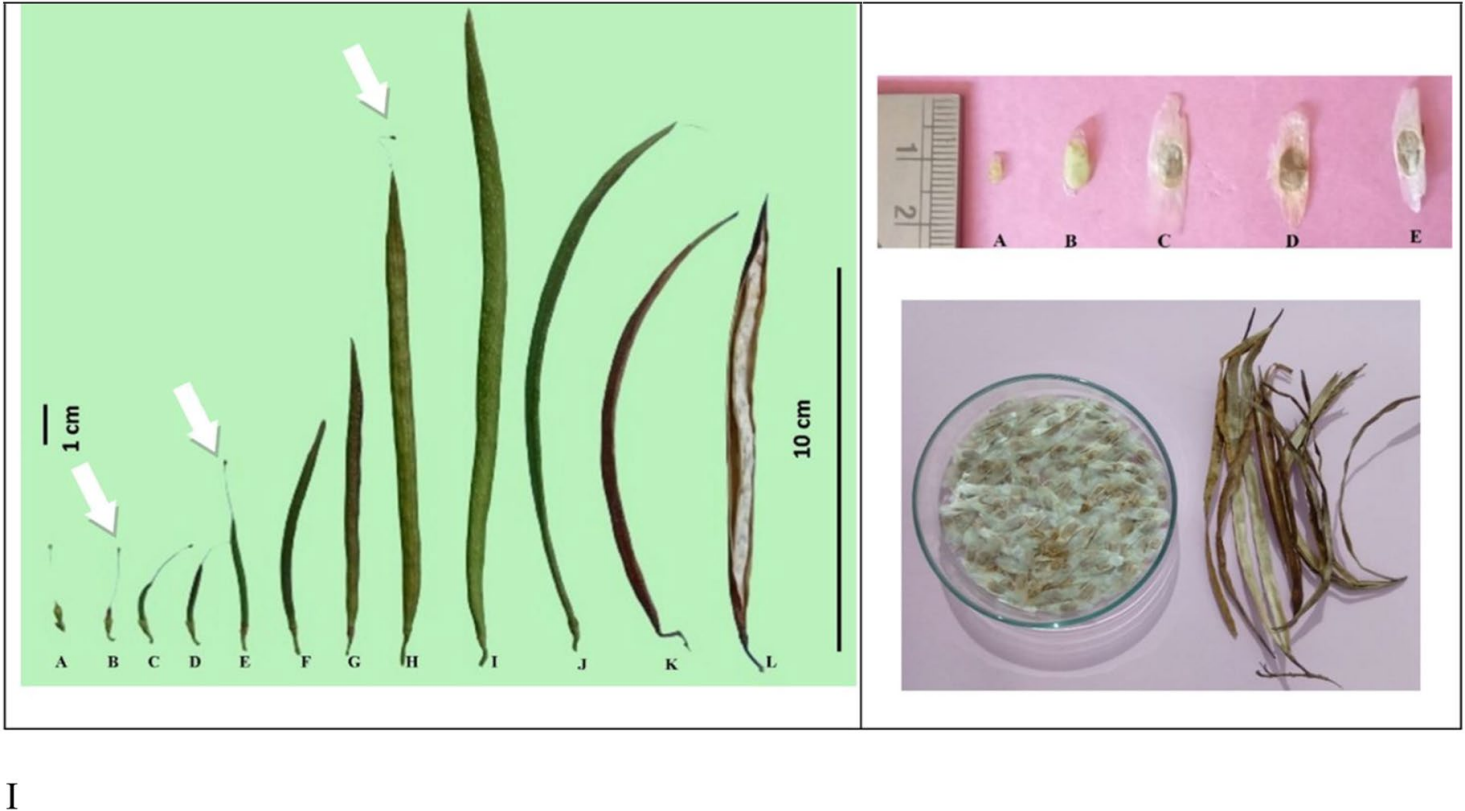
Stages of *Tecoma stans* pod development: (**AB**) Swell of ovary three days after fertilization I Visible fruit set (**D-E**) Pods began to lengthen longitudinally. (**F-G**) Appearance of constrictions indicating seed development (**H-I**) Pods expand to the maximum length (20 ± 2 cm) and width (1 cm) (**J-K**) Fully mature pods, constrictions disappear (**L**) Pods began to rupture from lateral sutures. Rana et al. 2023; Photograph cited with permission from Elsevier, license number 5,602,561,230,437

The shrub enters reproductive phase second year of its growth and begins flowering at a height less than a meter. In Cuba, the shrub has been seen blooming at a height of about 60 cm. However, it has been observed that the percentage of flowers yielding maturing fruits following fertilization success were found low in the *Tecoma* plants that came into bearing early, during second-third year of their growth (Anonymous 1958). A mean count of several old inflorescence on four shrubs growing on Mt. Long revealed that only 209 flowers yielded ripened fruits out of 1422 flowers, representing a fruit set of 14.7% ± 0.9% (Pelton 1964). The flowers which have not been successfully fertilized, remain attached by their persistent brown and dried pedicels on the non-woody rachis (Fig. 5). The pods initially green turn to pale brown and finally light grey on maturity and ripening. The seeds within pods are arranged overlapped and had wings on both elongated lateral sides giving a papery texture (Fig. 6; stage L). The mean fruit set of 18.1 ± 2.0% has been recorded from a single inflorescence. The ripened pods tend to rupture from their lateral sutures revealing overlapping seeds, however, these ripened pods can be seen retained on the shrubs for at least a year, due to absence of abscission layer at the junction of petiole of pod attachment with the branch.

The shrub exhibits asynchronous growth habit with the presence of leaves, flowers, developing immature and ripened pods at same period. *Tecoma* shrubs growing under the canopy of trees compete for air, sunlight and soil moisture that are limiting factors for their adequate flowering and fruiting. Plants growing under shade or that compete with other vegetation did not yield profuse flowers in an inflorescence or bear few flowers that fail to anthesis fully (Pelton 1964). Bor and Raizada (1954) found that *Tecoma* shrubs yielded better fruiting only in winter season, probably due to clear sunny weather. However, fruit yielding shrubs have been observed during summer and rainy season as well, though varying in intensity of fruit set. Seed set in *T. stans* is affected by several abiotic and biotic factors such as prevailing weather conditions (air temperature, relative humidity, wind speed, availability of pollinators etc.). Drought is considered as a major factor resulting in abscission of flower buds and failure of buds anthesis.

### Seed morphology and dispersal

The indehiscent ripened pods continue to disperse seeds via wind, until thoroughly weathered and may remain adhere to the branch in a cluster of vacant elongated brownish capsules. An average of 42 ± 9 seeds per capsule have been reported (Pelton 1964). The wind dispersal of seeds is common feature of several species from Bignoniaceae family. The seeds are dispersed through thin conspicuous translucent wings attached both side of the seed. The viable mature seeds have an average wings size of 24.2 ± 1.1 mm (maximum length) and 5.4 ± 0.2 mm (maximum width) (Fig. 5). The extremely thin whitish pair of wings are manifestations of the seed coat outgrowths that extend two-thirds of the length of the seed (67.4 mm + 2.9%). Perpendicular to their longitudinal axis, these wings form a narrow border, constituting 24.7 mm ± 1.9% of the total seed width. The wing margins usually appear fimbriated at their ends or sometimes even smooth (Pelton 1964).

In natural habitat, *Tecoma* shrubs colonize in form of dense stands, mostly in semi-open sites or often a times on exposed sites. Inhabitation in exposed sites is conducive for their seed dispersal through wind that allows the light winged seeds to drift away, even with a gentle breeze. Wind speeds (at Beaufort scale) exceeding gentle breeze at 12–19 kph (8–12 mph) tend to blow the winged seeds that may disperse several hundreds of miles away from the mother plant that could probably be the convincing reason for the widespread distribution of *T. stans*, particularly in the islands of West Indies (Grisebach 1864). It has been found that several regions surrounding Jamaica and the Central America are likely the initial source of the *Tecoma* species that later spread the seeds through high velocity winds to the remote regions over these islands. Plant explorers also maintain the opinion that *Tecoma* reached the United States into Florida through wind dispersal by seeds via the West Indies. Similarly, the widespread population of *Tecoma* progeny in Arizona is believed to be the seed dispersal from the *Tecoma* shrubs inhabiting in deserted regions of Mexico.

Although the seeds of *Tecoma* being light in weight, readily float on the surface of water for several days. However, it is still not clear if they retain their viability while being in contact with sea water and subsequent germination after dispersing on land. Seeds have been reported to occasionally be dispersed by streams, runoff, mammals, or birds.

### Seed germination

The seeds that are retained within ripened pods for extended period even after longitudinal dehiscence of capsules, have been found mostly non-viable due to repeated exposure to abiotic environmental stress factors across seasons, resulting either in seed rotting or may reveal poor seed germination. *Tecoma* seeds are ex-albuminous with conspicuous visible appearance of large foliaceouse marginate cotyledons through the semi-transparent coat (Fig. 5). Seed germination is epigeous with the appearance of seed leaves (cotyledons) 6th day after seed germination. Seeds of *T. stans* do not exhibit dormancy but possess a seed longevity of up to four years (Socolowski et al. 2008). Seeds (with visible embryos of more than one-half size) stored in airtight containers at room-temperature exhibited an 85% germination rate with a 7.2% moisture content even after four years of storage (author’s personal observation). Similar germination results (92.5 ± 2.1%) were observed by Rana et al. (2023). Further, it was found that the seeds sown at greater depth revealed poor germination. It has been observed that the seeds are photoblastic in nature, that respond to germination when exposed to incident light. In addition, the seeds placed over the soil surface and lightly covered with soil reveal better germination, relative to those sown at greater (40–80 mm) soil depth (Reis et al. 2020). Poor seed germination of *Tecoma* was recorded when seeds were sown in a finer textured clayey soil with relatively slow internal drainage compared to the seeds that were sown in porous well drained sandy loam soils. Seeds also exhibit variation in germination in wide range of temperature (< 20 ^o^C to over > 37 ^o^C), however, exclusion of light or sowing of seed at greater depth is a limiting factor. The seeds imbibed in water revealed peak germination rates within two days at room temperature, whereas the seeds that were simultaneously planted in the well-drained soil emerged the radicle in 8 days. A random sampling of *Tecoma* seeds from a lot (representative of both defective and normal appearing seeds), revealed up to 66.6 ± 3.6% germination. Defective seeds which are empty, with discolored embryos or smaller than half the size is not usually viable.

### Plant growth characteristics

*Tecoma* seedlings usually grow rapidly under favorable environmental conditions. The seeds of *Tecoma* may also germinate underneath and the area surrounding the *Tecoma* shrubs as a result of mature seed falling on the ground and the sapling often can be found in abundance during the monsoons. Under its native range the growth and development of seedlings is limited by prolonged drought or disturbance as a result of trespassing or grazing by herbivores. Seedlings develop a strong taproot that often penetrates the bedrock and cannot be excavated easily. Other factors such as presence of nearby vegetation causing shade to the developing seedlings, susceptibility of young saplings to fire are probably the limiting factors affecting the growth of seedlings. Color variation in terminal leaf primordia is evident due to varying environmental and edaphic factors. Often a times, anthocyanin pigments became conspicuous in leaf primordia prior to unfolding. As a consequence, the expanding terminal leaves reveal purplish tinge when sufficiently exposed to morning sunlight (author’s personal observation). Leaves at terminal growing shoots soon change to trifoliate pattern and later to compound leaves usually with 2 pair to leaflets with single terminal leaf. The photoperiod response and temperature regimes of *Tecoma* seedling finishing stages was optimized during greenhouse production of seedlings and it was found that *T. stans* nursery plants should be finished at temperatures 20^o^C or greater to avoid flower-bud abortion at cooler temperatures and improve flowering characteristics (Torres and Lopez 2011b).

### Growth pattern and natural regeneration

*Tecoma stans* usually is a micro-phanerophyte (any shrub or tree having a height of 2 to 8 m) with several stems usually emerging at least by a nodal distance of 15 cm. Occasionally a growing sapling develops a single or a standard trunk and usually began branching at ~ 0.5 m from the ground level (Pelton 1964). The trunk girth varies with age of the shrub; however, Seibert (1948) reports the trunk of old *Tecoma* shrubs reaching 25 cm in diameter in Mexico. *Tecoma* shrubs exhibit a moderately spreading growth habit, with vigorous shoots emerging from the nodes of arching branches. The emerging erect growing shoots aptly described species name ‘stans’ meaning ‘growing erect’. The growth pattern is asynchronous, partly form inflorescence terminating a vegetative shoot, and partly succeeding a vegetative growth developing from axillary buds. The leaves and buds are oppositely arranged. Each node bear two pairs of vegetative buds (one pair for leaf emergence and other for vegetative shoots).

The peripheral branches emerging from near the ground up to 30 cm height usually lean apart from the central axis and as the shoots become heavy, they begin to arch resulting in sprouting of side shoots (suckers) from the basal portion of the branches. *Tecoma* shrubs growing in their wild habitat may vary in height up to 7.1-8.0 m (Record and Hess 1940). On rare occasions, the height of shrub has been recorded up to 8–10 m in Peru (Macbride 1961). However, the rate of growth under optimum field conditions has been observed relatively higher as compared to its growth under natural habitat (Bruggeman 1957). The *Tecoma* variety *angustatum* is dwarf and is usually less than 1 m in height. *T. stans* does not often reproduce vegetatively under natural conditions, but instead sprouts vigorously from removed stumps or following a forest fire. Vigorously re-sprouting suckers from the ground were observed in recently burned areas (Gilman and Watson 1993). After stump pruning, *Tecoma* plants often develop sprouts from axillary buds after 18 days. Sucker shoots from the remaining stumps frequently grow to a height of 4.5 m but only have a breast-height diameter of 2 cm.

### Landscape use and cultural practices

**Fig. 7:**
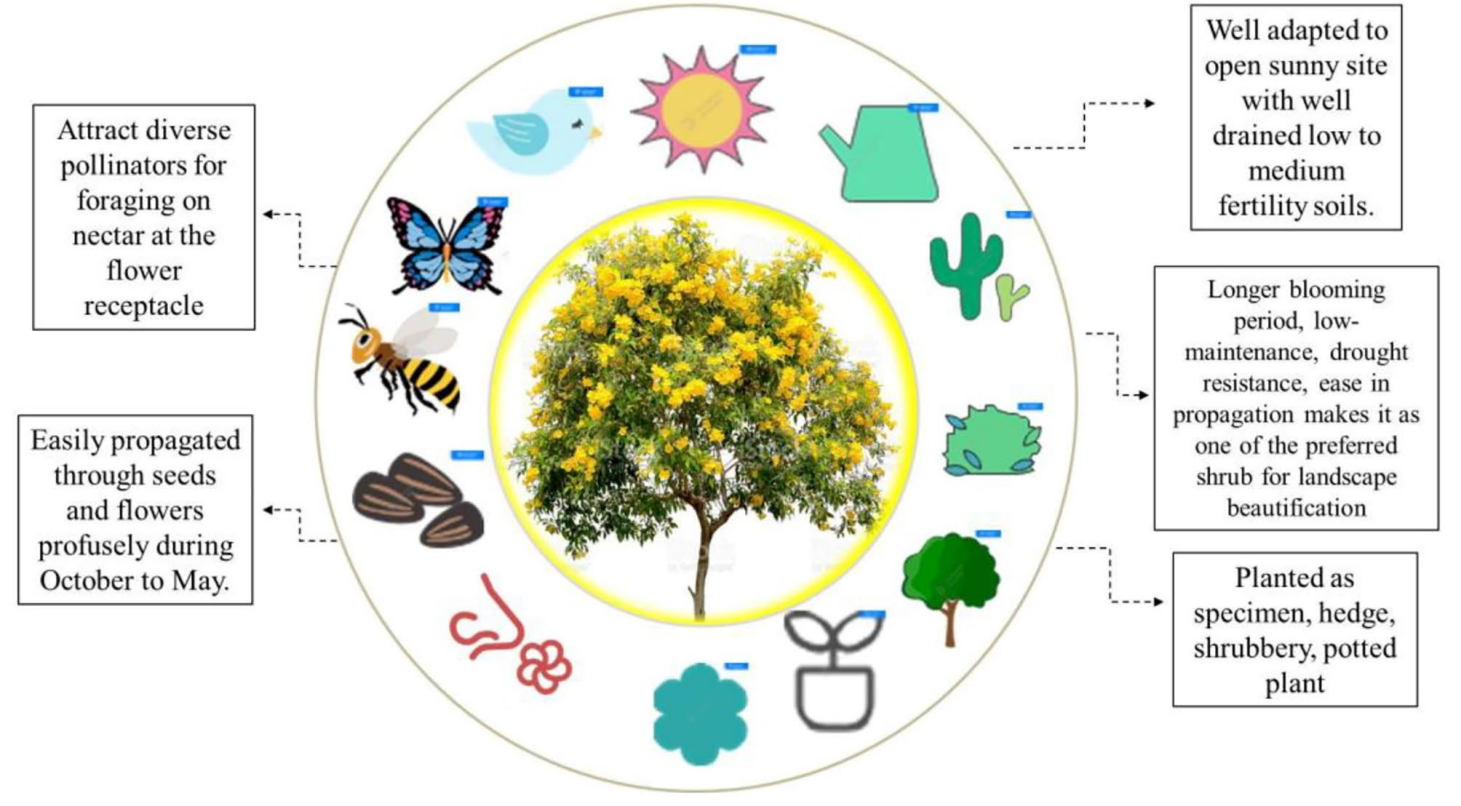
Representation of ecological, cultural aspects and ecosystem services of *Tecoma stans*

The Bignoniaceae has several tree, shrub, and herbaceous perennial species that are being utilized extensively in beautification of residential and public landscapes, while other species have promising ornamental worth. The prominent species in this family include flowering trees (*Jacaranda acutifolia* Bonpl., *Tabebuia aurea* Benth. and Hook.f. ex S.Moore, *Tecomella undulata* (Sm.) Seem., climbers (*Bignonia gracilis* G.Lodd., *Pyrostegia venusta* (Ker Gawl.) Miers,) and shrubs (*Tecoma castanifolia* (D.Don) Melch., *Tecoma fulva* (Cav.) G. Don, *Tecoma grandiflora* (Thunb.) Loisel., *Tecoma nyassae* (Oliv.) Baill. and *Tecoma stans* (L.) Juss. ex Kunth.) Amongst these species, *Tecoma stans* has a versatile use in landscape plantations as specimen, shrub border, potted flowering ornamental. Flowers attract diverse insects that pollinate the flowers and seek sweet nectar at the flower base, indicating its suitability for plantation in sensory gardens. *Tecoma* shrubs have the ability to adapt under excess soil moisture or water-logged conditions, though not for prolong period of time (Fig. 7).

The shrub needs a drastic pruning and can be maintained as dwarf tree less than 4.5 m in height. The pruning and pinching of the spent pods and flowers encourage branching, compactness and promotes recurrent blooming with peak flowering during March to April and September to November. Recurring blooming for longer duration, drought resistance, ease in sexual propagation with abundant seed formation in *Tecoma* makes it as one of the preferred yellow flowering shrubs for beautification of landscape and across extensive stretch of road dividers at highways (Pal and Krishnamurthi 1967).

### Therapeutic significance

Leaf extracts of *T. stans* are the chief source of bioactive components (monoterpene alkaloids, phenolic acids, flavonoids, and fatty acids) that contribute to its therapeutic benefits. A potential pancreatic lipase enzyme inhibitory drug comprising chrysoeriol and apigenin has been isolated from leaf extract (Ramirez et al. 2012). Tecomine and chlorogenic acid isolated from plant leaves have exhibited glucose-lowering ability (Rodriguez de Sotillo and Hadley 2002. These compounds are believed to possess nephrotoxic, antioxidant properties besides an effective antidote for urinary disorders (Minal and Namde 2014; Bakr et al. 2019). Costantino et al. (2003) reported that oral intake of flower infusion is beneficial for treatment of diabetes and stomach pains. *Tecoma* roots are a potential source of anthranilic acid and leaves contain several alkaloids such astamine and tecomine which are known to possess potential hypoglycemic effects (Al-Azzawi et al. 2012). The root-extracts have also been use in fermentation to make beer. Silver nanoparticles have also been synthesized by scientists using plant leaves and flowers, demonstrating its value in biomedicine, food packaging, and wound healing (Kumar et al. 2013). *Tecoma* leaf infusion has been used by Mexican scientists to treat hyperglycemia. Native Americans used plant components to treat a variety of illnesses that include hyperglycemia, intestinal and kidney problems. Leaf decoctions have been proved beneficial for cure of jaundice, skin infections, toothaches, headaches, joint pains and sore eyes. Leaf extracts have also been used for treatments for snake, scorpion, and rat bites (Anand and Basavaraju et al. 2019). In Mexico, the nectar forage by bees yields honey with a characteristic flavor.

At an industrial scale, this shrub has been used in extraction of various components like perfumes, cosmetics, flavouring and in manufacturing of lubricants. Methanolic leaf extract obtained from *T. stans* were found to possess anti-bacterial properties against *Streptococcus aureus* (Khatak et al. 2019). In a recent study, the crenatoside, phenylethanoid glycoside isolated from *T. stans*, exhibited a potent *in-vitro* antiviral activity against the *Zika virus* (Reis et al. 2020). Several in vitro and in vivo models related to obesity, hypoglycaemic and antihyperglycaemic effects have also been proposed (Aguilar-Santamaría et al. 2009; Ramírez et al. 2012). The compounds such as luteolin, apigenin, and chrysoeriol extracted from leaves through hydro-alcoholic maceration were reported as most active sub-fractions. Luteolin-rich organic fraction was found effective in treating metabolic syndrome alterations (Morales-Ferra et al. 2022).

### Response to biotic and abiotic stresses

The shrub is listed as a vulnerable species to root rots, *Clitocybe tabescens* in Florida and *Phymatotrichum omnivorum* in Texas (Westcott 1960). Physiological disorders were not commonly observed, except chlorosis of mature leaves over vigorous sprouts. Behavior of *T. stans* under drought conditions is strikingly similar to the characteristics exhibited by xeromorphic vegetation. The leaves of *T. stans* exhibit temporary wilting for four days under deficit soil moisture and leaves regain their turgor with availability of adequate soil moisture. However, the leaves may exhibit permanently wilting during an extended drought period and may fail to regain the turgor later even under rainfall. In very extreme drought situations, the leaf petioles can form abscission layers that enable leaf shedding during, and the shrub is often observed with few leaves, or may bear no leaves at all, and flowerless. Under short drought periods, the petioles droop and leaflets lose vigor, curl unevenly, and the tip of immature shoots appear pendant. With the emergence of black leaflet tips, the edges of the leaflet tend to dieback. On pollarded stumps, emerging suckers often wilt, although less severely, due to a greater root-to-shoot ratio. When exposed to higher temperatures, *T. stans* leaves partially loose vigour due to higher rate of transpiration. Nevertheless, the leaves of *Tecoma* have an exceptional physiological resilience to persistent wilting, which is less common in other mesomorphic species. Even during mild cooler air temperature during the dry season, the turgor pressure is not restored in the leaves; rather, they regain their vigor after a rainfall event. Do not often recover after light rainfall, most likely because absorbing feeder roots of *Tecoma* in its natural habitat are present deep within the limestone crevices. In Jamaica, *T. stans* has been referred as ‘deciduous’ (Asprey and Robbins 1953). In Australia, it has been observed with sparse foliage during winters (Smith 1894). However, *T. stans* is regarded as evergreen shrub in much of its ecological habitat range Menninger (1953). The presence of glandular hairs over the *Tecoma* leaves prevents the buildup of excessive hydrostatic pressure with the release of exudates through hydathodes and hence, protecting the plant from aeration stress resulted due to water-logged landscapes (Bor and Raizada 1990).

### Invasive potential and control

As noted in seed discussion, the potential exists for *Tecoma* to be invasive. The common saying ‘prevention is better than cure’ is very relevant for containing the invasive spread of *T. stans* before its further encroachment becomes problematic and unmanageable. The young germinating seeds or establishing seedlings can be removed manually or mechanically. It often exhibits quick and vigorous re-growth, when cut leaving behind the stubs; therefore, mechanical control is not much effective due to its quick resurgence from the leftover stumps.

#### Chemical control

There is little information available about the chemical control of this species. Oakes (1970) described an herbicidal formulation containing 2,4- D plus 2,4,5- T that was applied to the stem base to check its growth. It has been observed that effective control of this species is only achieved, when the herbicide has sufficiently translocated deep within the rhizosphere. Amino-cyclo-pyrachlor has been found effective herbicide to control the growth of *Tecoma*, besides suppressing the growth of annual and perennial weeds. However, this herbicide has a long-lasting effect on the soil. Amino cyclo-pyrachlor, being systemic in nature, is absorbed by the leaf epidermis and root hairs and subsequently translocated through vascular system, reaching plant meristes regions (Reis et al. 2015, 2016). Established *Tecoma* plants can be cut or eradicated before flowering followed by application of effective herbicides. Herbicides, triclopyr (4%), picloram (2%) and in combination as triclopyr + picloram (1%+1%) applied near the stump of *Tecoma* plants had shown 95% efficacy in containing the growth of *Tecoma* 270 days after application (Mendes et al. 2016). In another study, tebuthiuron granules applied near the base of the trunk was found effective at the application rate of 2 g per plant (Passini and Kranz 1997).

#### Bio-control

Mechanical and chemical control methods for *Tecoma* are not economically feasible as left-over stumps tends to re-surge, following reduction in efficacy of applied herbicides. Several bio-control agents have been evaluated and tested to curtail the further spread of this species. A bio-control programme was launched to check the invasion of *Tecoma* species from emerging as a weed in South Africa (Olckers 2004). An attempt was made to control the spread of *T. stans* through gall-forming rust fungus *Prospodium transformans*Ellis and Everh. Cummins (Pucciniales: Uropyxidaceae) and *Clydonopteron sacculana* Bosc, (Lepidoptera: Pyralidae) as a biological control agent (Wood 2014). Teliospores germination was found to occur at 18–22 °C. Despite easily inducing galls on plants grown in quarantine glasshouse conditions in South Africa, this rust fungus failed to establish itself in the field after being released. Later trials also confirmed the inefficacy of *Clydonopteron sacculana* Bosc in checking the growth of *Tecoma* shrubs. Another potential agent, *Dibolia sp.* (Coleoptera: Chrysomelidae), a flea beetle feeding on roots, failed to establish in quarantine after it was brought into South Africa (Madire et al. 2011). A decade later, evaluation of another biocontrol agent *Heikertingerella sp*. was carried out to check the invasion of *T. stans*. The larval and adult stages of the beetle caused significant damage to the root system and leaves of *T. stans*, respectively. The results concluded that this root-feeding flea beetle was found effective in predating the *Tecoma* shrubs and was recommended as a potential biocontrol agent for control of *T. stans* in South Africa (Madire and Netshiluvhi 2021; Madire et al. 2021). Two leaf-feeding agents, *Madapolluta* (Mulsant) (Coleoptera: Coccinellidae) and *Pseudona pomyza* sp. Hendel (Diptera: Agromyzidae), were subsequently released in South Africa in 2013 and 2014, respectively. *Mada polluta* successfully established at seven sites at lower elevation coastal regions of the Eastern Cape and KwaZulu-Natal provinces, but failed to establish at higher elevation inland areas. It was found that this beetle was host-specific for *Tecoma* and voraciously fed upon the species, reducing *Tecoma* spread. Later the beetle was recommended to serve as a plant growth control agent, reducing further invasion of *T. stans* in South Africa (Madire 2013). Apart from utilizing bio-control agents, *Tecoma* seedlings are vulnerable to herbivore browsing, which can lead to reduced seedling survival and establishment, and can serve as a check on potential invasiveness.

## Conclusion and future perspective

The review on *T. stans* highlights its comprehensive ecological distribution, morphology and reproductive biology, in addition to its pharmaceutical and landscape use, reported from India and worldwide. Due to its recurring bright yellow trumpet shaped flowers and retention of handsome foliage round the year, it is extensively used for landscape beautification as a shrub specimen, hedge, and also over highway road dividers due to its hardy and drought tolerant characteristics. *Tecoma* naturally regenerates from seeds and does not often reproduce vegetatively. However, if cut back, it can sprout aggressively from stumps. Widespread distribution of *Tecoma stans* has been credited to the wind dispersal of winged seeds to larger distances. Although, it is considered an invasive species in wastelands and grazing regions across Argentina, Australia, South Africa, Pacific Islands, Atlantic Islands and Asia, it is also an important nectar forage resource for the Apoidea family for nearly 48 species of bees. The act of nectar foraging saves *Tecoma* shrubs from herbivory due to presence of ants that seemingly annoy herbivores, which may be a positive attribute for its reproduction and survival. This species has been extensively investigated for used in pharmacological studies due to its therapeutic properties. Leaf extract contain several bioactive compounds that are believed to possess antioxidant, nephrotoxic, anti-microbial, antifungal, antibacterial and anti-proliferative properties. Invasive nature, encroachment of cultivated areas and interference with native species is the major concern of *T. stans*.

Considering its spectacular display of recurring yellow trumpet shapes flowers, ease of seed propagation, potential therapeutic benefits and an excellent source of bee-flora, this shrub offers much more positive aspects to consider. Plantings of *T. stans* can be a feasible solution for reclamation of waste and degraded lands, besides low maintenance landscapes. Besides cultivating *T. stans* as a promising flowering plant for landscape beautification, commercially, it can be cultivated for extraction of bioactive compounds such as monoterpene alkaloids, phenolic acids, flavonoids, carotenoids, terpenoids, glycosides, phytosterols, volatile oils, and unsaturated fatty acids that that contribute to its therapeutic benefits. Additionally, there is need to assess its complete nutritional and phytochemical profiling for further use in pharmacological and pharmaceutical studies for its therapeutic effects and delve deeper into its clinical relevance. Several studies undertaken on therapeutic benefits, makes it a potential plant to harness compounds of medicinal value, which could be a noble and novel way to utilize these aggressive growing plants for betterment of humanity and potentially reduce the negative perception of an ‘invasive’ plant to a ‘beneficial’ plant, across the regions worldwide in the prevention and treatment of diseases.

### Electronic supplementary material

Below is the link to the electronic supplementary material.

Supplementary Table S1. Morphological description of species of genus *Tecoma*.

## Data Availability

All sources of information are included in this review article. All data generated or analysed during this study are included.

## References

[CR1] Aguilar-Santamaría L, Ramírez G, Nicasio P, Alegría-Reyes CA, Herrera-Arellano (2009). Antidiabetic activities of *Tecoma stans* (L.) Juss. Ex Kunth. J Ethnopharmacol.

[CR2] Al-Azzawi AM, Al-Khateeb E, Al-Sameraei K, Al-Juboori AG (2012). Antibacterial activity and the histopathological study of crude extracts and isolated tecomine from *Tecoma stans* Bignoniaceae in Iraq. Pharmacogn Rev.

[CR3] Allen PH (1956). The rain forests of Golf o Dulce.

[CR4] Anand M, Basavaraju R (2019). Antimicrobial efficacy of *Tecoma stans* (L.) Juss ex Kunth: a review. Eur j Biotechnol Biosci.

[CR5] Anonymous (1958). Flowering plants from Cuban gardens.

[CR6] Apgar AC (1910). Ornamental shrubs of the United States.

[CR7] Arceo-Gomez G, Martinez ML, Parra-Tabla V, Garcia-Franco JG (2011). Anther and stigma morphology in mirror image flowers of *Chamaecrista chamaecristo ides* (Fabaceae): implications for buzz pollination. J Integr Plant Biol.

[CR8] Asprey GE, Robbins RG (1953). The vegetation of Jamaica. Ecol Monogr.

[CR9] Bailey LH (1941). The standard cyclopedia of horticulture.

[CR10] Bakr RO, Fayed MA, Salem MA, Hussein AS (2019). *Tecoma stans*: alkaloid profile and antimicrobial activity. J Pharm Bioallied Sci.

[CR11] Bertin RI (1982). Floral biology, hummingbird pollination and fruit production of trumpet creeper (*Campsis radicans*, Bignoniaceae). Am J Bot.

[CR12] BGCI (2019) Plant Search online database. Richmond, UK. https://www.bgci.org/plant_search.php. (Accessed: 17 September 2023)

[CR13] Bhat M (2019). Remedial and phytochemical review study on *Tecoma stans*. Int J Agric Sci.

[CR14] Bor NL, Raizada MB (1954). Some beautiful Indian climbers and shrubs.

[CR15] Bor NL, Raizada MB (1990) Some beautiful Indian climbers and shrubs. In: Bignoniaceae. BNHS Pp. 34–44

[CR16] Bridgewater S, Pennington RT, Reynel CA, Daza A, Pennington TD (2003). A preliminary floristic and phytogeographic analysis of the woody flora of seasonally dry forests in northern Peru. Candollea.

[CR17] Bruggeman L (1957). Tropical plants and their cultivation.

[CR18] CABI, Wallingford (2023) *Tecoma stans* (yellow bells). Invasive species compendium. CABI. 10.1079/cabicompendium.5295. Assessed on 10 May 2023

[CR19] Corner EJH (1940) Wayside trees of Malaya. Govern. Print. Office. Singapore. 2 vols

[CR20] Costantino L, Laura R, Renato P, Tiziana B, Pompeo P, Fabio G, Paulino Lins A, Daniela Barlocco L, Antolini, Samia A, El-Abady (2003). Isolation and pharmacological activities of the *Tecoma stans* alkaloids. Farmaco.

[CR21] Cruden RW (1977). Pollen-ovule ratios: a conservative indicator of breeding systems in flowering plants. Evolution.

[CR22] Cunningham Peter L (2008) *Tecoma stans* a potential invasive alien in Namibia? 30:33–39

[CR23] Curti RN, Ortega-Baes P (2011). Relationship between floral traits and floral visitors in two coexisting Tecoma species (Bignoniaceae). Plant Syst Evol.

[CR24] Dafni A (1992). Pollination ecology: a practical approach.

[CR25] East EM (1940). The distribution of self-sterility in the flowering plants. Proc Am Philos Soc.

[CR26] GBIF (2023) *Tecoma stans* (L.) Juss. ex Kunth in GBIF Secretariat. GBIF Backbone Taxonomy. Checklist dataset 10.15468/39omei accessed via GBIF.org. Accessed on 17 September 2023

[CR27] Gentry AH (1979). Distribution patterns of neotropical Bignoniaceae: some phytogeographical implications. Tropical botany.

[CR28] Gentry AH (1992) Bignoniaceae Part II (Tribe Tecomeae). Flora Neotropica. New York, USA: NYBG 285–290

[CR29] Giddy I (2004) Trees of Cloudbridge – *Tecoma stans* (Candelillo or Yellow elder). http://cloudbridge.org/trees/tecoma_stans.html

[CR30] Gilman EF (1993) DG Watson *Tecoma stans*: Yellow Elder Alien weeds and invasive plants. A complete guide to declared weeds and invaders in South Africa. Plant Protection Research Institute Handbook No. 12. Agricultural Research Council, Pretoria: pp. 300

[CR31] GISD Global Invasive Species Database (2023) Species profile: *Tecoma stans*. Downloaded from http://www.iucngisd.org/gisd/species.php?sc=1266 Accessed on 17 September 2023

[CR32] Goldblatt P, AH Gentry (1979) Cytology of Bignoniaceae. Botaniskanotiser 132:475–482

[CR33] Govindu HC (1950). Studies in the embryology of some members of the Bignoniaceae. Proc Indian National Sci Acad.

[CR34] Grisebach AHR (1864) Flora of the British West Indian Islands. London pp 24

[CR35] Harborne JB, Smith DM (1978). Anthochlors and other flavonoids as honey guides in the Compositae. Biochem Syst Ecol.

[CR36] Henderson L (2001) Alien weeds and invasive plants. Plant Protection Research Institute Handbook 7o. 12. Agricultural Research Council

[CR37] Hume EP (1951) Some ornamental shrubs for the tropics. U. S. Dept. Agr. Circ 34

[CR38] IPNI (2023) International Plant Names Index. https://www.ipni.org/p/1023-2. Accessed 8 January 2024

[CR39] IUCN, IUCN SSC Global Tree Specialist Group (2019) Botanic Gardens Conservation International (BGCI) and. *Tecoma stans*. The IUCN Red List of Threatened Species: e.T82858855A149060597. 10.2305/IUCN.UK.2019-3.RLTS.T82858855A149060597.en

[CR40] KBD, World Checklist of Vascular Plants (2023) Kew Backbone Distributions. The International Plant Names Index and 2023. Published on the Internet at http://www.ipni.org and https://powo.science.kew.org/ ©Copyright 2023 World Checklist of Vascular Plants. http://creativecommons.org/licenses/by/3.0

[CR41] Khatak S, Mali DK, Dahiya R (2019). *Tecoma stans*: a noxious weed put to beneficial use. Int J Chem Stud.

[CR42] Klein H (2002). Legislation regarding harmful plants in South Africa. PPRI Leaflet Series: weeds Biocontrol, no 1.2.

[CR43] Kobayashi N, Hagiwara JC, Miyajima I, Facciuto G, Soto S, Mata D, Escandon A (2004). A new pot plant variety bred by interspecific crossing between *Tecoma stans* (L.) HBK and *T. Garrocha* Hieron. J Jpn Soc Hortic Sci.

[CR45] Kumar R, Singh G (1988). Investigations into the cause of sterility *Tecoma stans* L. Bulletin of the Botanical Society of France. Bot Lett.

[CR44] Kumar A, Nima P, Astalakshmi A, Valuchamy G (2013). Green synthesis and characterization of silver nanoparticles using leaves of *Tecoma stans* (L.) Kunth. Int J Nanotechnol Appl.

[CR47] Macbride JF (1961). Bignoniaceae. In Flora of Peru. Field Museum of Natural History.

[CR48] Macdonald IAW, Nott TB (1987) Invasive alien organisms in central South West Africa/Namibia results of a reconnaissance survey. Madoqua15:21–34

[CR46] Madire LG (2013). Biology and host range of *Madapolluta*, a potential biological control agent of *Tecoma stans* in South Africa. Biocontrol Sci Technol.

[CR50] Madire LG, Netshiluvhi M (2021). Recent advances in the biological control of *Tecoma stans* L. (Bignoniaceae) in South Africa. Afr Entomol.

[CR51] Madire LG, Wood AR, Williams HE, Neser S (2011). Potential agents for the Biological Control of *Tecoma stans* (L.) Juss Ex Kunth Var. *Stans* (Bignoniaceae) in South Africa. Afr Entomol.

[CR49] Madire LG, Simelane D, Olckers T (2021). Pre-release evaluation of *Heikertingerella* sp. as a potential biocontrol agent for *Tecoma stans* in South Africa. J Appl Entomol.

[CR52] Mendes RR, Biffe DF, Constantin J, de Oliveira Júnior RS, Rosa ÊL, Cuba ALF, Baladeli RB (2016). Yellow bells (*Tecoma stans*) control in pasture with localized applications of herbicides. Revista Brasileira De Herbicidas.

[CR53] Menninger EA (1953). Catalog of flowering tropical trees.

[CR54] Menninger EA (1962). Flowering trees of the world for tropics and warm climates.

[CR55] Minal W, Namde H (2014). Callus induction studies and active components and antioxidant activity investigation from leaves and callus of *Tecoma stans* (L.) Juss. Ex Kunth. Res J Pharm Biol Chem Sci.

[CR56] Morales-Ferra DL, Zavala-Sánchez MÁ, Jiménez-Ferrer E, González-Cortazar M, Zamilpa A (2022). Effect of *Tecoma stans* (L.) Juss. Ex Kunth in a murine model of metabolic syndrome. Plants.

[CR57] Moza MK, Bhatnagar AK (2007). Plant reproductive biology studies crucial for conservation. Curr Sci.

[CR58] Muller F (1868). Befruchtungsversuchean Cip6 alho (Bignonia. Bot Zeit.

[CR59] Neal MC (1948). Gardens of Hawaii.Special Pubi. 40, Bernise P.

[CR60] Newcombe FC (1922). Significance of the behaviour of sensitive stigmas. Am J Bot.

[CR61] Newcombe FC (1924). Significance of the behaviour of sensitive stigmas II. Am J Bot.

[CR62] Oakes AJ (1970). Herbicidal control of *Tecoma stans* (L.) Juss. Ex HBK. Turrialba.

[CR63] Olckers T (2004). Targeting emerging weeds for biological control in South Africa: the benefits of halting the spread of alien plants at an early stage of their invasion. S Afr J Sci.

[CR64] Orwa C, Mutua A, Kindt R, Jamnadass R, Anthony S (2009) Agroforest tree Database: a tree reference and selection guide version 4.0. http://www.worldagroforestry.org/sites/treedbs/treedatabases.asp. Accessed on 10 May 2023

[CR65] Pal BP, Krishnamurthi S (1967) Flowering shrubs.pp.122. Krishan Kumar, New Delhi

[CR66] Passini T, Kranz WM (1997). Herbicides efficiency on trumpet flower (*Tecoma stans*). Planta Daninha.

[CR67] Pelton JA (1964). Survey of the ecology of *Tecoma* stans. Butl Univ Bot Stud.

[CR69] Pennington RT, Prado DE, Pendry CA (2000). Neotropical seasonally dry forests and quaternary vegetation changes. J Biogeogr.

[CR68] Pennington RT, Lavin M, Prado DE, Pendry CA, Pell SK, Butterworth CA (2004). Historical climate change and speciation: neotropical seasonally dry forest plants show patterns of both tertiary and quaternary diversification. Philos Trans Royal Soc.

[CR70] Pesman MW (1962) Meet flora Mexicana. Dale King. Pubi. Globe. Arizona.pp.278

[CR71] Petersen C, Brown JH, Kodric-Brown SA (1982). An experimental study of floral display and fruit set in Chilopsislinearis (Bignoniaceae). Oecologia.

[CR72] PIER Pacific Island Ecosystems at Risk (2023) *Tecoma stans* (L.) Juss. ex Kunth. http://www.hear.org/pier/species/tecoma_stans.htm. Accessed 17 September 2023

[CR73] POWO Plants of the World Online (2023) *Tecoma stans* (L.) Juss. ex Kunth. https://powo.science.kew.org/taxon/urn:lsid:ipni.org:names:111284-1. Accessed on 10 May, 2023

[CR74] Prado DE, Gibbs PE (1993). Patterns of species distribution in the dry seasonal forests of South America. Ann Mo Bot Gard.

[CR75] Ramirez G, Zavala M, Perez J, Zamilpa A (2012) *In vitro* screening of medicinal plants used in Mexico as antidiabetics with glucosidase and lipase inhibitory activities. Evidence-based complementary and alternative medicine 1–6. 701261. 10.1155/2012/10.1155/2012/701261 PMC3469274 23082084

[CR76] Rana N, Singh S, Dhakad AK, Dhatt KK (2023). Coding phenological growth stages of yellow bells (*Tecoma stans* (L.) Juss. Ex Kunth) based on BBCH scale and its implications for urban greening. Curr Plant Biol.

[CR77] Rao S, Aluri JSR, Victor P (2005). Steady state flowering pattern, temporal dioecism, facultative xenogamy and pollination by insects in *Tecoma stans* L. (Bignoniaceae). J Natl Taiwan mus.

[CR78] Record SJ, Hess RW (1940). American timbers of the family Bignoniaceae. Trop Woods.

[CR81] Reis FCD, Tornisielo VL, Cason JB, Dias ACR, Freitas M, Sotomayor JF, Filho RV (2015). Uptake, translocation, and control of trumpet flower (*Tecoma stans*) with aminocyclopyrachlor. J Environ Sci Health.

[CR80] Reis FC, Cason JB, Toledo REB, Sotomayor JF, Freitas MM, Filho RV (2016). Aminocyclopyrachlor: New Option for Trumpet Flower Control in pastures. Planta Daninha.

[CR79] Reis ACC, Silva BM, de Moura HMM, Pereira, Brandão GC (2020). Anti-zika virus activity and chemical characterization by ultra-high performance liquid chromatography (UPLC-DAD-UV-MS) of ethanol extracts in Tecoma species. BMC Complement Med Ther.

[CR82] Rodriguez de Sotillo DV, Hadley M (2002). Chlorogenic acid modifies plasma and liver concentrations of: cholesterol, triacylglycerol, and minerals in (fa/fa) Zucker rats. J Nutr Biochem.

[CR83] Sbihi HM, Mokbli S, Nehdi IA, Al-Resayes SI (2015). Physico-chemical properties of *Tecoma stans*. Linn. Seed oil: a new crop for vegetable oil. Nat Prod Res.

[CR84] Scogin R (1980). Anthocyanins of the Bignoniaceae. Biochem Syst Ecol.

[CR85] Seibert RJ (1948). The use of glands in a taxonomic consideration of the family Bignoniaceae. Ann Mo Bot Gard.

[CR86] Silva CI, Augusto SC, Sofia SH, Moscheta IS (2007) Diversidade de abelhasem *Tecoma stans* (L.) Kunth (Bignoniaceae): importâncianapolinização e produção de frutos [Bee diversity in *Tecoma stans* (L.) Kunth (Bignoniaceae): importance for pollination and fruit production]. Neotrop Entomol 36:331– 41. 10.1590/s1519-566x2007000300002. PMID: 17710317 10.1590/s1519-566x2007000300002 17710317

[CR87] Singh S (2023) Biometeorological influence on the phenology of Yellow Bells (*Tecoma stans* L.) and implications on reproductive success. In: Abstracts of 23rd International Congress of Biometeorology, Arizona State University, USA, 14–17 May 2023

[CR88] Smith E (1894) *Tecomasmithii* X. Gardeners’ Chron. 16:64

[CR89] Socolowski F, Vieira DCM, Takaki M (2008). Interaction of temperature and light on seed germination in *Tecoma stans* L. Juss. Ex Kunth (Bignoniaceae). Braz Arch Biol Technol.

[CR90] Standley PC (1926). Trees and shrubs of Mexico. Contrib United States Natl Herbarium.

[CR91] Suryakanta (1973). Pollen morphological studies in the Bignoniaceae. Rev Palaeobot Palynol.

[CR92] Taher MA, Dawood DH, Sanad MI, Hassan RA (2016). Searching for anti-hyper glycemicphytomolecules of *Tecoma stans*. Eur J Chem.

[CR93] Torres AP, Lopez RG (2011). Photosynthetic daily light integral during propagation of *Tecoma stans* influences seedling rooting and growth. Hort Sci.

[CR94] Torres AP, Lopez RG (2011). Photoperiod and temperature influence flowering responses and morphology of *Tecoma stans*. Hort Sci.

[CR95] USDA ARS (2013) National Genetic Resources Program. Germplasm Resources Information Network (GRIN). Online searchable database. http://www.ars-grin.gov/gringlobal/search?q = Tecoma%20stans

[CR96] Vasey G, Rose JN (1890). List of plants collected by Dr. Edward Palmer m Lower California and western Mexico in 1890. Contr U S Natl Herb.

[CR97] Walker-Larsen J, Harder LD (2000). The evolution of Staminodes in Angiosperms: patterns of Stamen Reduction, loss, and functional re-invention. Am J Bot.

[CR98] Westcott C (1960) plant Disease Handbook. Van Norstrand. New York. 825 p

[CR99] Wojcik VA (2011). Bees (Hymenoptera: Apoidea) utilizing *Tecoma stans* (L.) Juss. Ex Kunth (Bignoniaceae) in Urban landscapes: a comparison of occurrence patterns and community composition in Three cities in Northwestern Costa Rica. J Kans Entomol Soc.

[CR101] Wood JRI (2006) Inter-Andean dry valleys of Bolivia – floristic affinities and patterns of endemism: insights from Acanthaceae, Asclepiadaceae and Labiatae. In: Pennington RT, Ratter JA, Lewis GP eds. Neotropical savannahs and seasonally dry forests. Systematics Association Special 69: 235–56

[CR100] Wood AR (2014). Observations on the gall rust fungus *prospodium transformans*, a potential biocontrol agent of *Tecoma stans* var. *Stans* (Bignoniaceae) in South Africa. Trop Plant Pathol.

