# Supplementary material for: A comprehensive review on ecology, life cycle and use of Tecoma stans (bignoneaceae)
Source: Bot Stud. 2024 Feb 13;65:6. doi: 10.1186/s40529-024-00412-4 (PMC10861415; doi:10.1186/s40529-024-00412-4)
Supplement: Supplementary file 1 — Supplementary Table S1. Morphological description of species of genus Tecoma. [file 40529_2024_412_MOESM1_ESM.docx]

**Supplementary** **Table S1**. Morphological description of species of genus *Tecoma*

| **Sr.No** | **Species/Common Name** | **Distribution** | **Growth habit/Branches** | **Leaves** | **Inflorescence** | **Fruits and seeds** |
| --- | --- | --- | --- | --- | --- | --- |
|  | *Tecoma stans*  (The yellow elder, Trumpet flower, Yellow bells) | Native to Southern Florida and southern most Arizona | Shrub sometimes reaching the size of small tree 10m tall and 25 cm, the bark dark, ridged with lepiodote and somewhat subpuberulous branchlets | Leaves are opposite, compound and odd-pinnate 4-8 in. long serrate and lanceolate | Corolla yellow (occasionally slightly orange-yellow) with reddish lines in throat | Seeds 3-5 mm long, 2.4-2.7 cm wide, the wings hyaline-membranaceous, sharply demarcated from seed body |
|  | *Tecoma arequipensi syn. Pandorea pandorana sub spp Pandorana* | Indigenous to Southern Peru and Northen Bolivia | Shrub, 1-4 m tall, branchlets glabrous, slightly lepidote | Pinnate, 5-15 foliolate, serrated leaflets petiolate | Orange-red, Terminal raceme | Linear capsule, tapering at ends, calyx persistant, 6-10cm long, 6-10mm wide, Seeds thin, bialate with wings hyaline-membranaceous |
|  | *Tecoma australis* / *Pandorea pandorana* (Wonga-wonga vine) | Indigenous to Australia | Climber with cylindrical and striate stem | Compound, odd pinnate and exstipulate sessile, smooth and glabrous | Highly scented, corolla with purple throat. | Does not produce fruits |
|  | *Tecoma capensis*Lindl./  *Tecomaria capensis*  (The cape honeysuckle) | Native to South Africa and adjacent southernmost Mozambi | A rambling shrub about 2 m tall. Stem and branches slender, brown with prominent raised lenticles. | Compound with terminal leaflet, membranaceous, | Corolla red to orange red, slightly curved, mostly glabrous and ciliated lobes. | Capsule, subtended by the persistent calyx, the surface slightly minutely lepidote,, wrinkle- ridged. |
|  | *Tecoma castanifolia*  (Chestnut leaf trumpetbush) | Endemic to the dry part of coastal Ecuador, mostly from north western coastal Peru. | 4 or 5 m tall Shrub | Simple trifoliate, serrate 4-20 cm long 1-7 cm wide leaves | Cupular calyx, corolla red or orange,red with yellow orange throat | Fruit a linear capsule, tapering to ends, subterete when fresh, seeds thin, with hyaline-membranaceous wings, sharply demarcated from seed body |
|  | *Tecoma cochabambensis* | Native to central Bolivia | Shrub with glabrous branchlets | Leaves are pinnate, with serrate leaflets, lanceolate to narrowly oblong eliptical | Cupular calyx, with red- orange corolla. | Linear capsule, tapering at ends with calyx persistant Seeds with the wings hyaline membranaceous, sharply demarcated from seed body. |
|  | *Tecoma fulva*  (Chuve) | Native to Atacama Desert region of northern Chile and southernmost coastal Peru | Shrub with sparsely puberulous branchlets | Leaves pinnate with small the leaflets, obovoid serrate with broadly triangular teeth in apical half, cuneate to rounded base and coriaceous. | Yellow to orange corolla tube with red or red-orange lobes, tubular-infundibuliform,, tube completely glabrous outside and inside | Capsule, tapering at ends with seeds thin, bialate, 5-7 mm long, 20-25 mm wide, the wings hyaline membranaceous, sharply demarcated from seed body. |
|  | *Tecoma garrocha*  (Orange trumpet bush) | Native to the Americas | Shrub or small tree 2-5 m tall, the branchlets slightly lepidote inconspicuously and minutely puberulous | Leaves pinnate with leaflets remotely serrate, lanceolate to narrowly acutish, | Deep yellow-orange to orange colour of the flowers, born in clusters on the terminals of new branches. | Linear capsule, tapering at ends with thin bialate seeds |
|  | *Tecoma grandiflora/ Campsis grandiflora*  *(*Chinese trumpet creeper) | Indigenous to China and Japan | Climber with few or no aerial roots with angled, smooth and glabrous stem | Leaves are opposite, compound and odd pinnate with petiole, often unequal sided at the base, coarsely toothed, glabrous on both surface leaflets | Scarlet or orange corolla, with marked scarlet striation in the throat. Inflorescence terminal pendulous with racemose panicles | Capsule not beaked at the apex |
|  | *Tecoma guarume* (Cahuato) | Native to Peru | Shrub with glabrous branchlets | Pinnate, shallowly serrate, elliptic to obtanceolate, apically obtuse basally cuneate, ,long glabrous except for inconspicuous lepidote scales. | Red to red-orange corolla tube with orange to yellow-orange lobes and usually a yellow throat with 1-1.5 cm long basal tube | Linear capsule with bialate seeds having hyaline-membranaceous wings demarcated from seed body. |
|  | *Tecoma jasminoids/ Pandorea jasminoides*  (The bower plant of Australia) | Indigenous to Australia | Climbing shrub with terete stem | Narrow leaflets with elliptic, ovate or lanceolate in shape, sessile, blunt at the tips, dark green in colour, glabrous, 1-1.5 in. long | White or rosy pink corolla with dark pink or almost purple the hairy throat Inflorescence axillary or terminal many flowered corymbose panicles | Oblong capsule with wide winged seeds |
|  | *Tecoma nyassae*  (Trumpet bush) | Native to Tropical East Africa | Small tree | Ovate, very shallowly serrate, and even the lower pairs of leaflets usually acute or acutish, | Bilabiate corolla with a shorter tube and the reflexed lower lobes about as long as the tube | Smooth surface with wrinkled-striate fruit. |
|  | *Tecoma radicans* (Trumpet-vine) | Indigenous to North America. Commonly cultivated in gardens throughout India | Deciduous shrub, climbing by means of adventitious roots with cylindrical, smooth and polished branches emitting roots where they touch the soil. | Compound, odd-pinnate, opposite, leaflets | Orange corolla with a scarlet limb Inflorescence terminal clusters or panicles | Fruit an elongated angled capsule, 3-5 in. long, curved and beaked at the apex. Many seeds, compressed with two large, translucent wings |
|  | *Tecoma rosifolia*  (Fresno, fresnillo, hadda) | Native to Northern Andean Peru. | Shrub with the puberulous branchlets | Pinnate, 5-9 foliolate, serrate, elliptic to elliptic-ovate, apically obtuse to sub-acuminate leaflets | Orange or red orange corolla with orange yellow throat with reddish lines. Inflorescence terminal raceme | Fruit a linear capsule, tapering at ends, the calyx persistent. Seeds thin, bialate, with hyaline membranaceous, sharply demarcated wing |
|  | *Tecoma sambucifolia*  (Trumpet flower) | Native to Andes of Peru and southernmost Ecuador | Shrub with glabrous branchlets except for a few inconspicuous lepidote scales to puberulous. | Leaves pinnate, l-3 foliolate, the leaflets closely serrate, elliptic, apically obtuse to more or less acute. | Yellow corolla with reddish penciling in throat. Inflorescence a terminal raceme | Seeds thin, bialate, 7-10 mm long, 15-27 mm wide, the wings hyaline membranaceous, sharply demarcated from seed body. |
|  | *Tecoma tenuiflora*  (Guaranguaycillo) | Southern Bolivia and northwestern Argentina | Shrub, with branchlets slightly lepidole to minutely puberulous | Leaves pinnate 3-5-13 foliolate, the leflets sharply and closely serrate with the tooth tips oriented towards leaflet apex, elliptic, apically acute to acuminate basally cuneate to rounded | Inflorescence terminal raceme | Seeds thin, bialate, 5-7 mm long, 12-15 mm wide, the wings hyaline membranaceous, sharply demarcated from seed body. |
